# Retraction Note: STK4 protein expression pattern follows different trends in endometrioid and serous endometrial adenocarcinoma upon tumor progression

**DOI:** 10.1038/s41598-026-63116-8

**Published:** 2026-07-21

**Authors:** Igor Govorov, Sanaz Attarha, Larysa Kovalevska, Emil Andersson, Elena Kashuba, Miriam Mints

**Affiliations:** 1 Division of Obstetrics and Gynecology, Department of Women’s and Children’s Health, Karolinska University Hospital, Solna, Karolinska Institutet, 171 77 Stockholm, Sweden; 2 Science for Life Laboratory, Karolinska Institutet, 171 65 Stockholm, Sweden; 3 R.E. Kavetsky Institute of Experimental Pathology, Oncology and Radiobiology of NAS of Ukraine, Kyiv, 03022 Ukraine; 4 Department of Microbiology, Tumor and Cell Biology (MTC), Karolinska Institute, Biomedicum, 17165 Stockholm, Sweden; 5 School of Medical Science, Faculty of Medicine and Health, Örebro University, 70182 Örebro, Sweden

Retraction of: *Scientific Reports* 10.1038/s41598-022-26391-9, published online 22 December 2022

The Editors have retracted this Article.

After publication, concerns were raised that Supplemental Figure 1A, D, E and F had previously been published in^1^ representing different data. The Authors did not respond to the Editors request for to the raw data. The Editors therefore have lost confidence in this Article.

Elena Kashuba disagrees with the retraction. Igor Govorov, Sanaz Attarha, Larysa Kovalevska, Emil Andersson, and Miriam Mints did not respond to the correspondence from Editors about this retraction.
